# Magnetic Resonance Imaging of Primary Hepatic Malignancies in Patients With and Without Chronic Liver Disease: A Pictorial Review

**DOI:** 10.7759/cureus.1539

**Published:** 2017-08-04

**Authors:** Lauren F Alexander, Peter Harri, Brent Little, Courtney C Moreno, Pardeep K Mittal

**Affiliations:** 1 Department of Radiology and Imaging Sciences, Emory University School of Medicine; 2 Department of Radiology and Imaging Sciences, Emory University School of Medicine

**Keywords:** liver neoplasms, carcinoma, hepatocellular, cholangiocarcinoma, magnetic resonance imaging, hemangioendothelioma, epithelioid, hemangiosarcoma, lymphoma, bile duct neoplasms

## Abstract

Primary hepatic malignancies are less common than metastatic diseases, but a recognition of these lesions is important for diagnosis and treatment planning. Magnetic resonance imaging (MRI) provides the most imaging information to diagnose lesions noninvasively and to narrow differential diagnoses. This paper reviews the imaging findings of chronic liver disease and primary hepatic malignancies, including hepatocellular carcinoma (HCC), intrahepatic cholangiocarcinoma (CCA), epithelioid hemangioendothelioma, hepatic angiosarcoma, and primary hepatic lymphoma. Clinical and MRI features are reviewed to improve the readers’ recognition of these tumors, allowing for a narrower differential diagnosis when liver masses are encountered on abdominal imaging.

## Introduction and background

With the increased use of medical imaging, liver lesions are found in both symptomatic and asymptomatic patients. Primary hepatic lesions are less common than metastatic diseases; however, their recognition is important for diagnosis and treatment planning. While ultrasound and computed tomography (CT) scans can identify liver lesions, magnetic resonance imaging (MRI) provides the most imaging information to diagnose lesions noninvasively and to narrow the differential diagnosis.

Primary hepatic malignancy is the second leading cause of death from cancer worldwide and is the fifth most common type of cancer in men and the ninth in women [1]. Within the United States, from 2003 to 2013, mortality due to primary liver cancer has increased at the highest rate compared to all other cancers in men and women [2]. The rise in chronic liver disease has increased the population at risk for hepatocellular carcinoma (HCC), which can be diagnosed by imaging features alone if the correct protocol is followed, decreasing the need for biopsy or other interventions. While multiphasic CT and MRI scans have similar sensitivity for lesions over two cm, an MRI scan provides higher soft tissue contrast, allowing for a more detailed assessment of each lesion’s internal characteristics as well as secondary features to confirm malignancy. For patients undergoing frequent surveillance for lesions, MRI avoids the potential radiation risks from serial multiphasic CT exams [3-4]. This paper reviews the MRI appearance of primary hepatic malignancies in patients with and without a chronic liver disease to improve the readers’ recognition of these tumors and to narrow the differential diagnosis when liver masses are encountered on abdominal imaging.

## Review

MRI Technique

A single MRI protocol, including magnetic resonance cholangiopancreatography (MRCP), can adequately evaluate pathology involving the liver, the biliary tree, and other solid organs of the abdomen. The routine abdominal MRI protocol should include precontrast axial and coronal T1-weighted (T1W) three-dimensional (3D) gradient echo (GRE) images with fat saturation; axial and coronal T2-weighted (T2W) single-shot fast spin echo (ssT2) images without fat saturation; axial ssT2 images with fat saturation; axial dual echo gradient echo images (in- and opposed-phases); axial steady state free precession images; and heavily T2-weighted fast spin echo MRCP (Table 1). Patients receive intravenous gadobenate dimeglumine (0.05 mmol/kg) for T1W 3D GRE dynamic contrast-enhanced images with fat saturation. Axial arterial phase T1W images are timed with a bolus tracking method, triggered when the contrast bolus reaches the aorta at the diaphragmatic hiatus. Appropriate arterial phase timing is critical to detect small tumor foci. The portal venous phase is obtained in the axial plane 70 sec post injection. Delayed images are obtained three minutes (axial) and five minutes (coronal) post injection. Dynamic contrast enhancement is essential to characterize liver masses, as discussed in detail below. In patients with diminished renal function, who may or may not require dialysis, a careful assessment of the risks and benefits of gadolinium-based contrast agents should be performed. Resources such as the ACR Manual on Contrast Media provide guidance for clinicians to determine whether or not these agents can be used for their patients [5]. Studies investigating the administration of gadobenate dimeglumine in patients with renal dysfunction found no cases of nephrogenic systemic fibrosis, so this agent could be considered in the appropriate setting to ensure complete evaluation when necessary [6-8].

**Table 1:** MRI protocol for evaluation of the liver MRI = magnetic resonance imaging, 2D = two dimensional, 3D = three dimensional, CP = cholangiopancreatography, GRE = gradient echo, PAT = parallel acquisition technique, SPAIR = spectral adiabatic inversion recovery, SSFSE = single-shot fast spin echo, SSFP = steady state free precession, TE = echo time, TR = repetition time

Sequence	Plane	Breath Hold	Fat Saturation	FOV (mm)	Matrix	# of sections	Thickness (mm)	Gap (mm)	TR (msec)	TE (msec)	Flip Angle (degree)	PAT Factor
T2 SSFSE	Axial, Coronal	No	No	350	256×198	35	7	0.7	1500	83	180	2
T2 SSFSE SPAIR	Axial	No	Yes	350	256×198	35	7	0.7	1500	83	180	2
SSFSE 2D MRCP	Axial	No	Yes	300	384×269	35	7	0.7	1500	685	180	2
MRCP 2D Slab	Coronal	Yes	Yes	300	384×269	1	50	N/A	4500	756	180	2
T1 3D Spoiled GRE	Axial, Coronal	Yes	Yes	380	288×200	88	3	N/A	3.8	2	10	2
T1 2D In- & Opposed-Phase GRE	Axial	Yes	No	380	320×256	35	6	1.5	150	2.2, 4.5	70	2
SSFP	Axial	No	No	380	256×256	50	6	0	3.07	1.54	70	2

Chronic Liver Disease

The world over, chronic liver disease is most commonly caused by chronic hepatitis B or chronic hepatitis C, nonalcoholic fatty liver disease, and alcoholic liver disease. Whatever the inciting cause, the resultant hepatocyte injury activates portal fibroblasts, which leads to the generation of collagen matrix materials. This process stimulates a cascade of portal fibrosis replacing the hepatic parenchyma. Consequently, the liver transforms into diffuse regenerative parenchymal nodules surrounded by fibrous bands and variable degrees of portosystemic vascular shunting [9-10].

**Figure 1:**
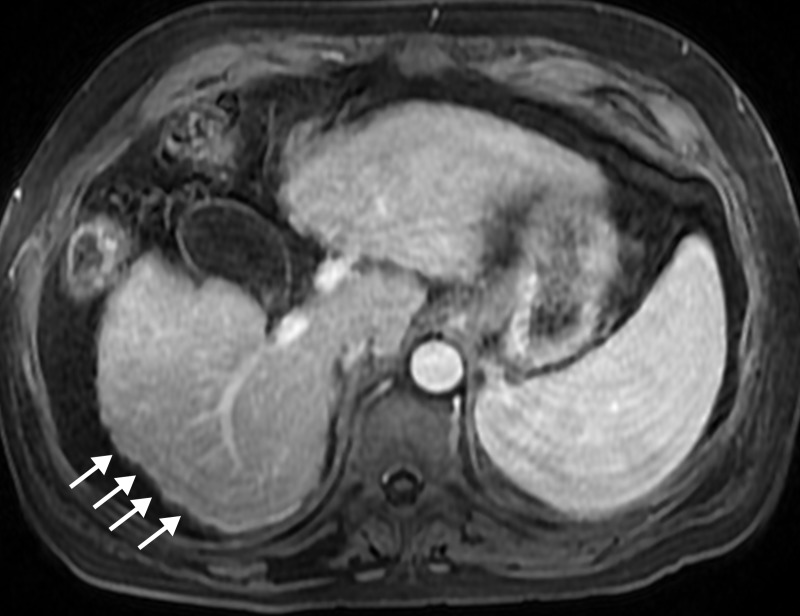
Cirrhotic liver The portal venous phase axial T1 fat saturation (FS) image shows hepatic surface nodularity (arrows) and a widening of the gallbladder fossa.

On imaging, patients with chronic liver disease demonstrate variable degrees of abnormal hepatic morphology, which can be readily identified on MRI scans. The liver parenchyma has a nodular appearance due to regenerative nodules and bridging fibrosis. The hepatic parenchyma volume is redistributed with caudate and left lobe hypertrophy, atrophy of the right lobe, and widening of the gallbladder fossa (Figure 1) [11].

**Figure 2:**
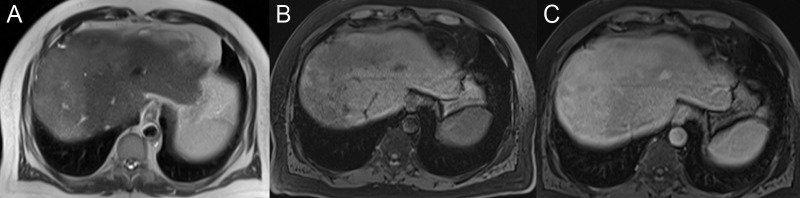
Hepatic fibrosis A: The axial T2 image shows hyperintense bands in the hepatic dome. B: The axial T1 weighted image with fat saturation (FS) shows that the bands have a hypointense signal before contrast. C: Delayed post-contrast axial T1 FS shows a delayed enhancement of the fibrotic bands.

If there is acute chronic inflammation, the liver may have patchy areas of heterogeneous arterial enhancement that persist or become isoattenuating on later phases. This enhancement is differentiated from focal lesions by a lack of defined margins or any corresponding signal abnormality on the precontrast sequences. Fibrosis can be identified as T2 hyperintense bands with fine linear or coarse reticular appearance and corresponding progressive delayed enhancement (Figure 2) [12].

**Figure 3:**
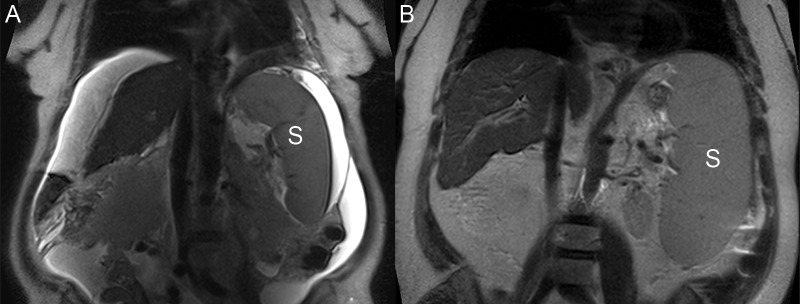
Imaging features of portal hypertension A: Coronal T2 image shows hyperintense ascites around the nodular liver and around the enlarged spleen (S). B: Coronal T2 image shows an enlarged spleen (S) filling the left upper quadrant.

Portal hypertension develops due to increased resistance to portal blood flow, which may be at the prehepatic (i.e., portal vein thrombus), intrahepatic (i.e., cirrhosis), or posthepatic (i.e., elevated right heart pressure) level. In the setting of chronic liver disease, portal hypertension is most commonly due to the underlying cirrhotic change causing increased resistance to portal venous flow [9]. Imaging findings include a dilated or diminutive main portal vein, an occluded main portal vein, and cavernous venous collaterals at the liver hilum. Portal hypertension results in splenomegaly, portosystemic varices (often with a recanalized umbilical vein), and ascites to variable degrees (Figure 3) [11-12].

Hepatocellular Carcinoma

The most common malignant liver lesion in the setting of a chronic liver disease is hepatocellular carcinoma (HCC); however, these patients are also at increased risk for cholangiocarcinoma (CCA) and combined HCC–CCA. The hepatocarcinogenesis sequence accounts for the development of HCC. As the hepatocytes regenerate to replace cells lost to necrosis and apoptosis, deoxyribonucleic acid (DNA) mutations cause nodules to become dysplastic and eventually malignant. As these nodules undergo malignant transformation, tumoral neovascularity transforms the nodule blood supply from primarily portal venous supply to a predominantly hepatic arterial supply, and these arterial vessels are often abnormal [4,9]. This process of malignant degeneration occurs across a continuum, and the diffuse regenerative nodularity of the liver parenchyma that occurs during cirrhosis can make the identification of small lesions challenging if a meticulous imaging technique is not used. The American Association for the Study of Liver Diseases (AASLD) [13] recommends surveillance using an ultrasound every six months for patients with cirrhosis of any cause or for patients with chronic hepatitis B, even without findings of cirrhosis. It is challenging to distinguish small HCC from regenerative nodules by ultrasound. Nodules less than one cm can be followed with more frequent ultrasounds, but nodules that grow or measure greater than one cm should be further characterized with contrast-enhanced cross-sectional imaging such as MRI [14].

**Figure 4:**
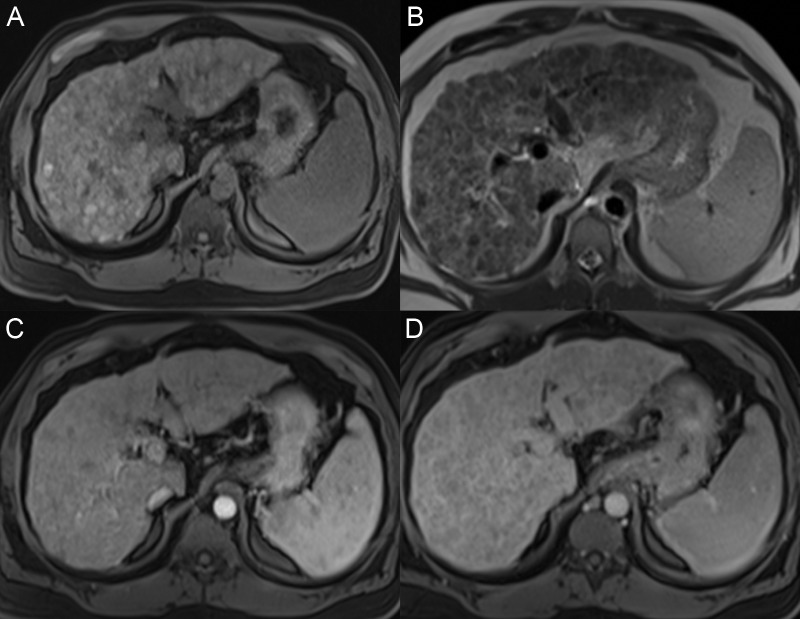
Regenerative nodules A: The axial T1 fat saturation (FS) image shows multiple nodules throughout the liver that are hyperintense compared with the liver parenchyma. B: On the axial T2 image, the nodules have a T2 signal similar or slightly darker than the liver. C: On the axial T1 FS arterial phase, there is no nodule enhancement. D: Nodules are only seen on the axial T1 FS delayed phase due to the delayed enhancement of the surrounding fibrotic tissue bands.

Unlike many malignancies, HCC can be diagnosed by its imaging features without the need for histologic confirmation if an appropriate imaging technique is utilized. The administration of intravenous contrast is critical to diagnosis, and multiple phases of contrast timing are required for the complete characterization of the liver mass enhancement pattern. Regenerative nodules tend to have a T1 signal intensity similar to or brighter than the adjacent parenchyma and the T2 signal same as the liver. The nodules are usually not well identified after contrast because they enhance the same as the surrounding liver unless there are surrounding fibrotic bands with delayed enhancement (Figure 4).

**Figure 5:**
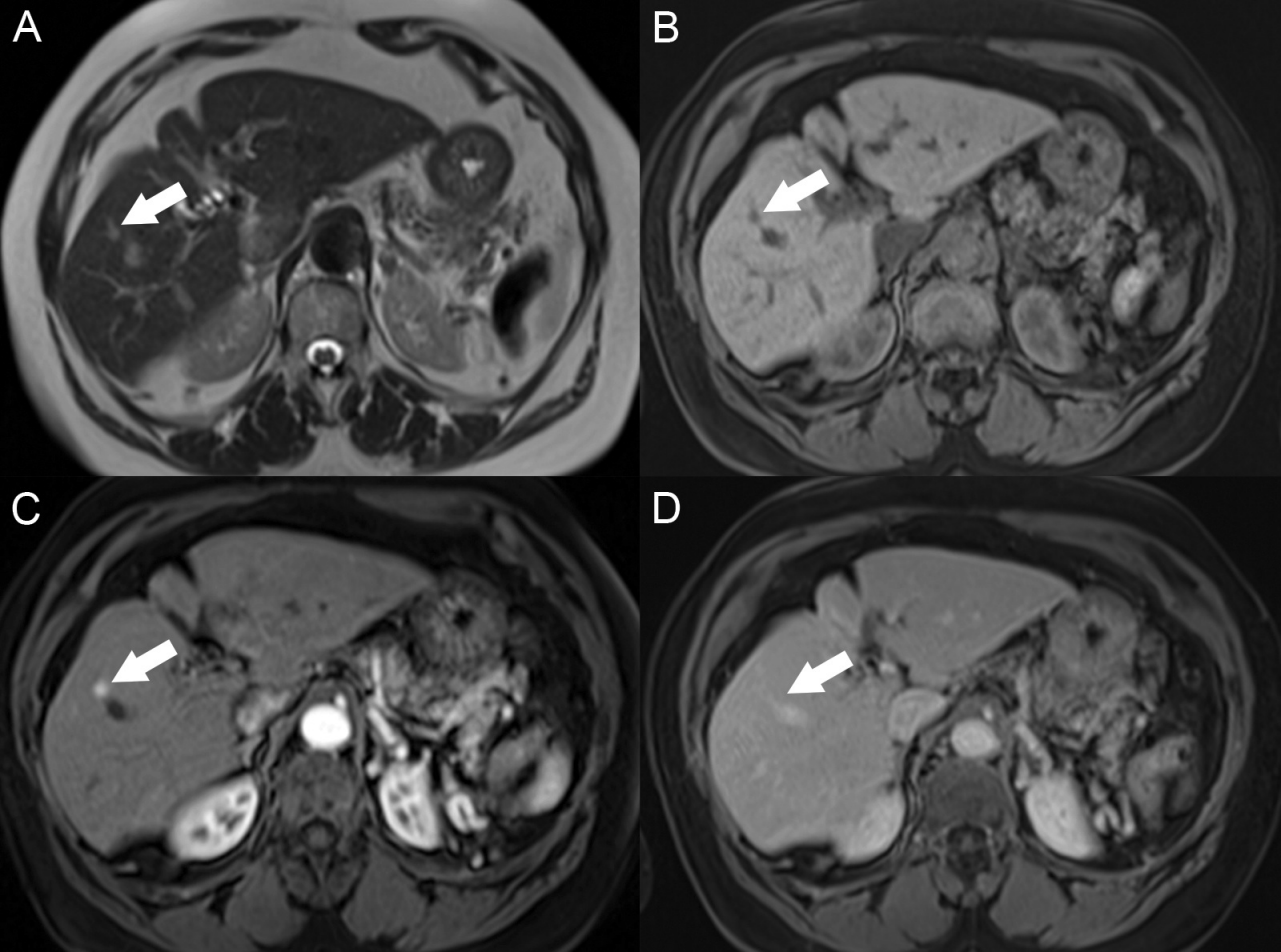
Dysplastic nodule in a cirrhotic liver A: The dysplastic nodule in the cirrhotic liver shows a hyperintesne signal on axial T2 (arrow). B: There is hypointense signal on axial T1 fat saturation (FS) (arrow). C: After contrast, the nodule has increased enhancement on arterial phase T1 FS (arrow). D: No washout in the nodule on delayed phase T1 FS (arrow).

Dysplastic nodules are considered a premalignant lesion and are differentiated by increased enhancement during the arterial phase, but there are no T2 signal changes, washout, or pseudocapsules on later phases (Figure 5). A short-interval follow-up in three months is recommended in these patients to monitor for further development into HCC [4,15].

**Figure 6:**
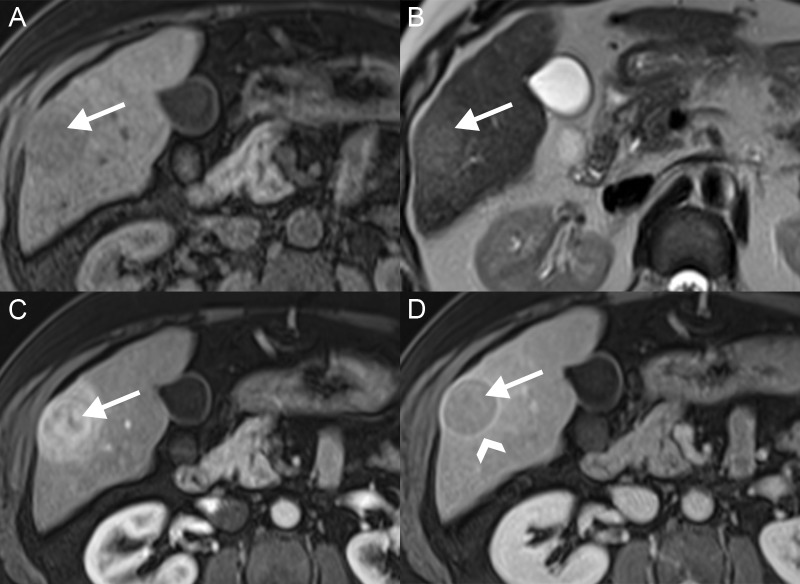
Classic hepatocellular carcinoma A: The lesion (arrows) is hypointense on axial T1 fat saturation. B: The lesion has a mildly hyperintense signal on axial T2. C: The lesion has avid enhancement on the T1 FS arterial phase. D: The lesions have washout on the T1 FS delayed phase with an enhancing pseudocapsule (arrowhead).

Three enhancement features are used to diagnosis HCC: arterial hyperenhancement, portal venous and/or delayed washout, and late enhancing rim or pseudocapsule (Figure 6). The most important imaging feature to diagnosis HCC is the presence of increased lesion enhancement during the arterial phase. At this point in time, the abnormal arterial neovascularity to the lesion results in increased contrast flow to the tumor, while the rest of the liver supplied by the portal venous system is not yet enhanced. The lesion enhancement is generally homogeneous but may be heterogeneous or rim-like [16]. The “washout” appearance, with decreased enhancement as compared to the adjacent liver parenchyma, is seen on the venous and delayed phases. In some cases, the lesion may be isointense on the portal venous phase, with washout only visible on the delayed phase [17]. The pseudocapsule is identified as a peripheral enhancement around the rim of the lesion on the portal venous and/or delayed phase [4,18].

**Figure 7:**
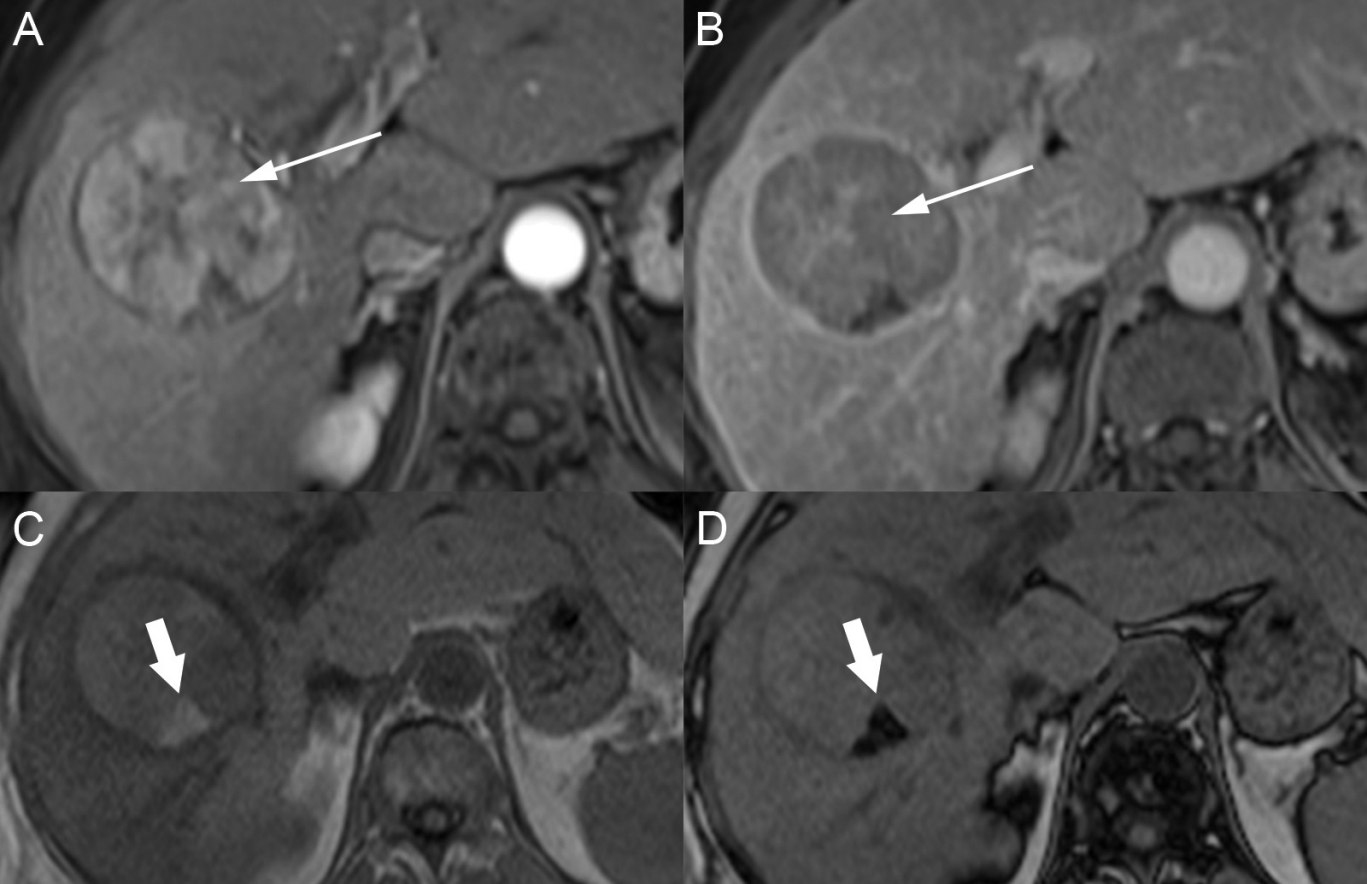
Hepatocellular carcinoma with microscopic fat A: The mass in the right lobe of the liver (thin arrow) has avid enhancement on axial T1 fat saturation (FS) in the arterial phase. B: On the T1 FS delayed phase, the mass has washout and pseudocapsule (thin arrow). C, D: The axial dual gradient echo in phase (C) and opposed phase (D) show a signal change from bright on the in phase to dark on the opposed phase (thick arrows), confirming intralesional fat.

Other imaging features can help identify HCC but are not part of the imaging definition. Lesions tend to have mildly increased (bright) T2 signal intensity but this signal change can be difficult to identify in cases with diffuse hepatic parenchymal heterogeneity. Some lesions contain areas of intracellular lipid, easily confirmed on the dual gradient echo sequence (Figure 7).

On diffusion imaging, HCC tends to have a bright signal in high b value series with a corresponding dark signal on apparent diffusion coefficient maps, which is consistent with restricted diffusion. These sequences can be prone to artifacts from ascites or patient motion and have reduced yield in patients with a significant iron deposition in the liver. Current AASLD guidelines do not take into account the role of hepatobiliary contrast agents that have both extracellular properties and uptake and excretion by hepatocytes, allowing for delayed hepatobiliary phase imaging [4,18].

In addition to identifying and characterizing the lesion as HCC, the hepatic vasculature should be carefully inspected for thrombus. Microvascular invasion is a finding of advanced HCC in pathologic specimens that are not readily apparent in current imaging studies; however, a progression to macrovascular invasion with malignant thrombus can be identified. The tumor can invade both the portal and hepatic vein branches in the region of the tumor and grow along the vessels towards the liver hilum or the inferior vena cava (IVC). This tumor should not be confused for a bland thrombus in the setting of portal hypertension. A tumor thrombus is usually contiguous with the malignant lesion and has irregular arterial phase enhancement. A bland thrombus should never enhance, and the presence of any enhancing thrombus in a vessel should raise concern about a tumor thrombus. Subtraction sequences can be helpful to bring out subtle enhancement [18].

**Figure 8:**
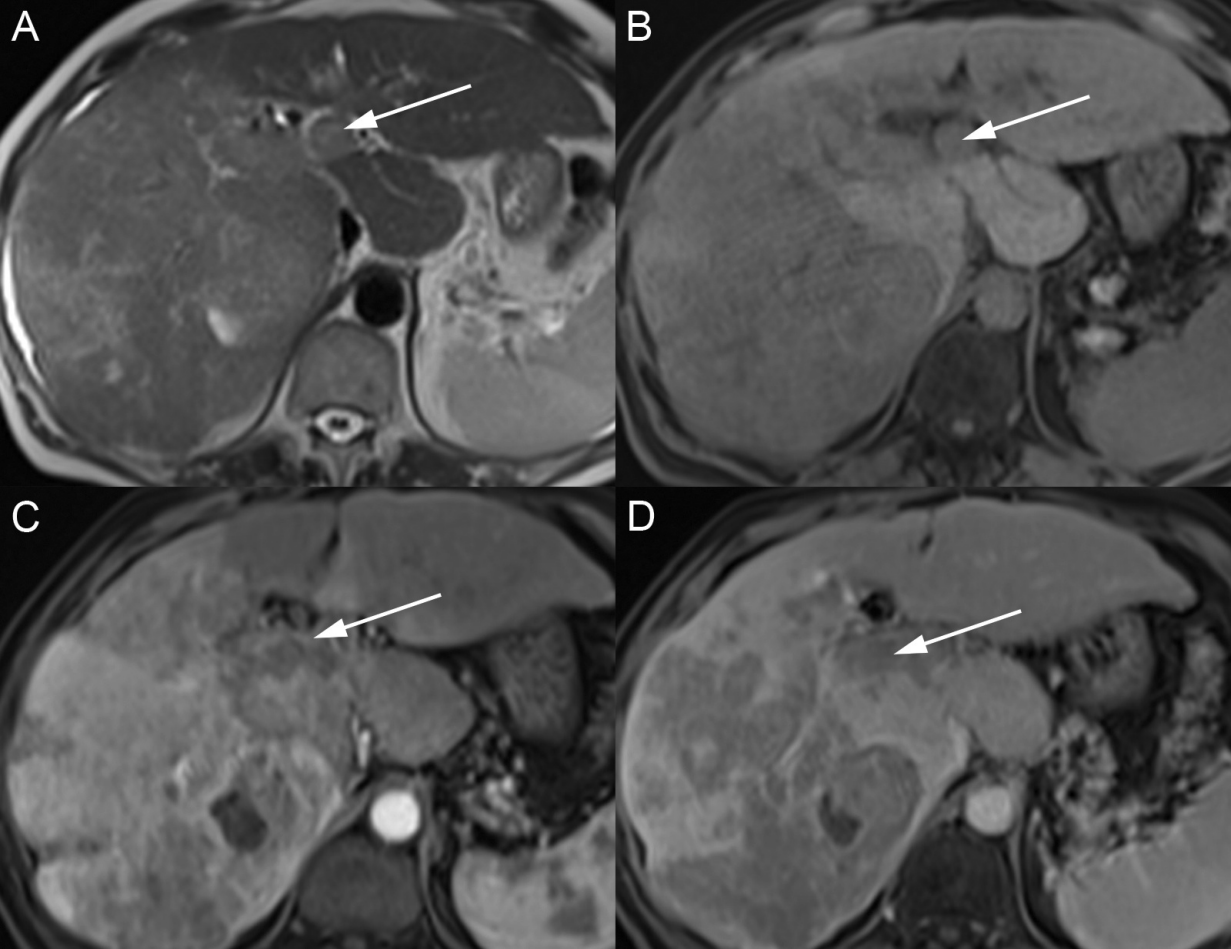
Infiltrative hepatocellular carcinoma with tumor thrombus A. The infiltrative hepatocellular carcinoma in the right hepatic lobe has heterogeneous, patchy increased signal intensity on axial T2. B. The mass has heterogeneous dark signal intensity on axial T1 fat saturation (FS). C: The mass has heterogeneous enhancement on axial T1 FS arterial phase D: The axial T1 FS delayed phase shows washout in the mass. The main portal vein tumor thrombus (arrows) shows similar precontrast signal changes, arterial enhancement, and washout to the tumor.

While HCC is often mass forming with discrete lesions visible on imaging, the liver must be carefully assessed for infiltrative HCC (Figure 8). This pattern of the tumor can be difficult to identify due to the heterogeneous background parenchymal changes of the chronic liver disease. The tumor has ill-defined, heterogeneous signal intensity, often mildly to moderately hypointense on T1W and mildly to moderately hyperintense on T2W. Arterial phase enhancement may be patchy, micronodular, or absent. On later phases, the tumor has variable degrees of washout. Tumor extension into the portal venous system occurs often with this form of HCC [19-20].

An MRI scan provides staging information in addition to diagnosis. Multiple staging systems have been proposed for HCC. The Barcelona Clinic Liver Center (BCLC) staging system is recommended by the AASLD because it considers the imaging features for staging as well as the clinical parameters of liver function and performance status. These combined features are used to guide treatment. Five different stages are described by the system, four of which include radiologic findings. The clinical factors are beyond the scope of this paper, but briefly, these components assess liver function with the Child-Turcotte-Pugh score and patient performance status with the Eastern Cooperative Oncology Group (ECOG) scale recommendations [14,21-23]. 

**Figure 9:**
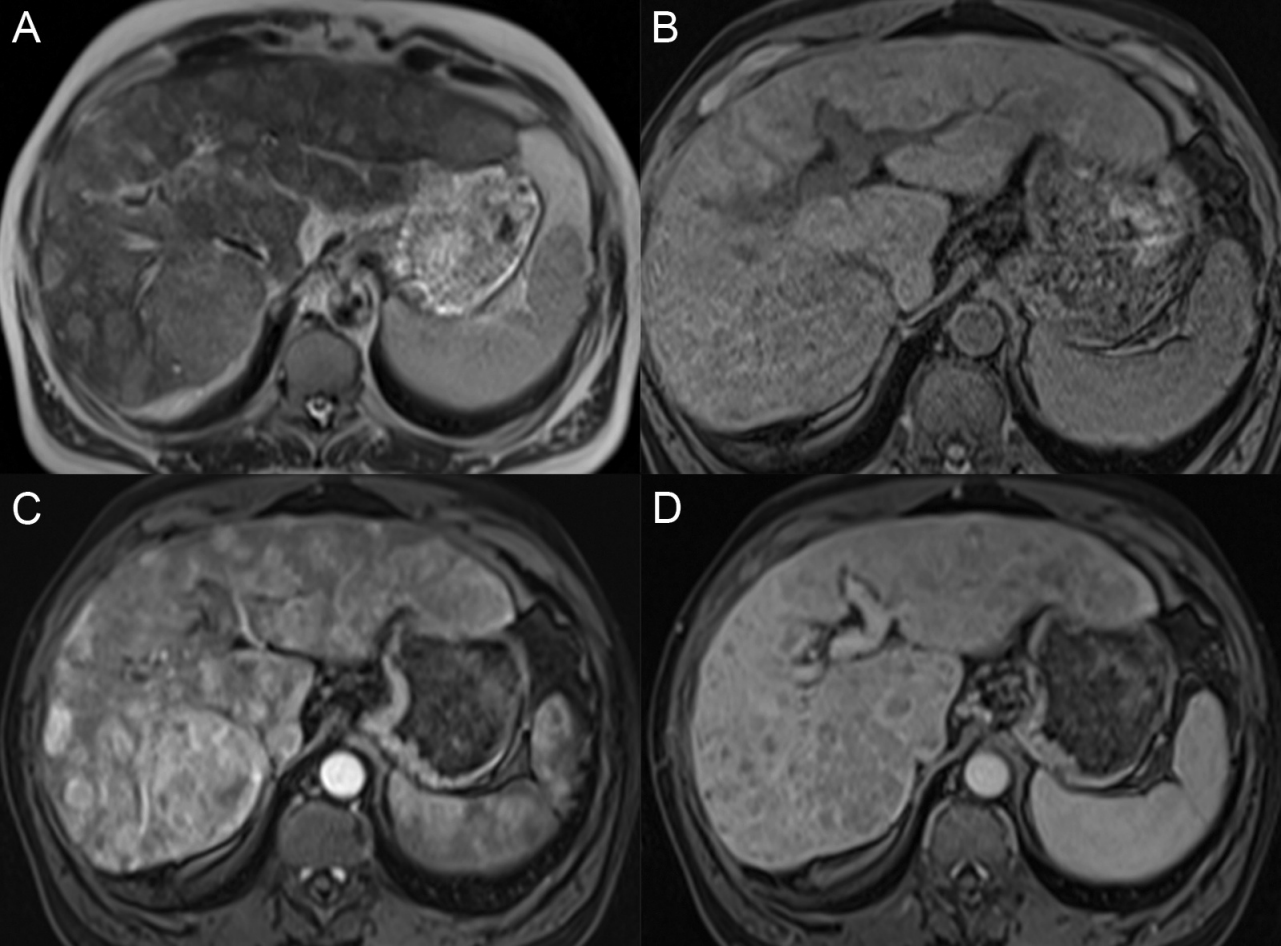
Multifocal hepatocellular carcinoma A: Numerous hyperintense lesions throughout the liver on axial T2. B: The lesions are hypointense on axial T1 fat saturation (FS). C, D: The lesions have avid arteial enhancement on axial T1 FS and washout on axial T1 FS delayed phase.

Radiologic BCLC stage 0 (very early stage) consists of a single HCC less than or equal to 2 cm in size. These lesions have extremely low chance of vascular invasion or satellite lesions. These patients can be managed with targeted resection or liver transplant, depending on bilirubin and portal pressures. Radiologic BCLC stage A (early stage) consists of a single HCC greater than two cm or up to three lesions, each less than three cm in size (Figure 6). If the patient falls under the Milan criteria of one lesion less than five cm or three lesions less than three cm, liver transplantation is a treatment option [24]. Patients who do not meet these criteria or who have other clinical factors may undergo resection or targeted ablation therapy. Radiologic BCLC stage B disease (intermediate stage) includes patients with a multifocal disease with a lesion greater than three cm or more than three lesions, without vascular invasion or extrahepatic spread (Figure 9). These patients can be treated with chemoembolization if they have appropriate liver function and performance status.

Radiologic BCLC stage C disease (advanced stage) has HCC with a vascular invasion of any type and/or extrahepatic spread (Figure 8). These patients can receive palliative therapy with sorafenib, an oral multikinase inhibitor, with a proven survival benefit [25]. BCLC stage D does not have specific radiologic features because these patients have poor liver function and performance status by the Child-Turcotte-Pugh score and the Eastern Cooperative Oncology Group scale [14,21-23]. 

HCC can occur in patients without an underlying chronic liver disease, particularly in patients with chronic hepatitis B infection, metabolic syndrome, and aflatoxin B exposure. The distribution is bimodal in the second and seventh decades with less male predominance than in cases with chronic liver disease. The MRI features are the same as classic HCC, but tumors tend to be larger and the disease more advanced, in part because these patients may not be included in surveillance protocols [26-27]. Because of the increased risk of HCC, patients with chronic hepatitis B should be undergoing routine surveillance.

Fibrolamellar Variant of HCC

**Figure 10:**
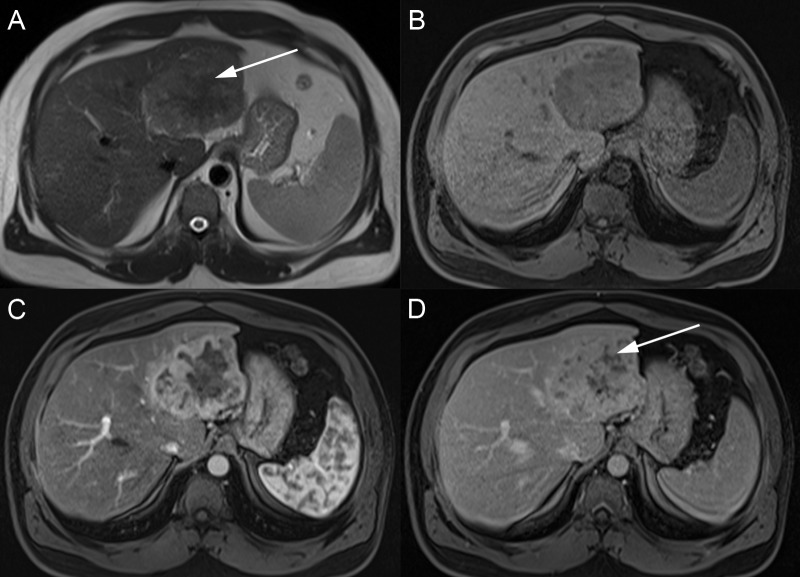
Fibrolamellar hepatocellular carcinoma A: The left hepatic lobe mass is mildly hyperintense on axial T2. B: The mass is hypointense on axial T1 fat saturation (FS). C, D: After contrast, the mass has heterogeneous enhancement on the axial T1 FS arterial (C) and delayed (D) phases. The mass has a T2 hypointense, hypoenhancing scar (arrows).

The fibrolamellar variant of HCC also arises in patients without cirrhosis, and these patients are usually less than 40 years' of age (average age: 23-25 years) without any risk factors for a chronic liver disease. The pathway for the development of these tumors is not well known, but they are pathologically different from HCC with prominent fibrous tissue and a dense central scar. Alpha-fetoprotein (AFP) levels are usually normal [28-29]. At imaging, these masses are usually hypointense on T1WI and hyperintense on T2WI but the central scar remains dark on both T1WI and T2WI due to fibrosis. Fat has not been reported in fibrolamellar HCC, but calcifications can be present. On post-contrast T1WI, the mass usually has heterogeneous arterial phase enhancement with variable washout or isoattenuation in the portal venous and delayed phases (Figure 10). Enhancement of the central scar is variable, but it is generally hypoenhancing on the post-contrast phases [30].

Cholangiocarcinoma

Malignancies arising from the biliary tree are the second-most common type of hepatic malignancy after HCC and develop from the epithelial lining of the bile ducts [29]. CCA accounts for 10%-15% of all primary hepatic malignancies. Risk factors include processes with biliary inflammation, including primary sclerosing cholangitis, liver fluke infection (Opisthorchis viverrinia and Clonorcis sinesnsis), hepatolithiasis, cirrhosis, and bile duct anomalies (Caroli’s disease and choledochal cysts). Incidence is highest in southeast Asia, where liver flukes and hepatolithiasis are most common, but the incidence is increasing in North America, and CCA is now the leading cause of primary liver tumor death in the United States. Unlike HCC, many of the risk factors are less easily prevented [31-33].

The tumors are classified by intrahepatic or extrahepatic location, with extrahepatic tumors further subdivided into hilar and distal categories. The majority of tumors are extrahepatic, slightly more in the hilar location than the distal extrahepatic bile duct (50% vs 40%). Tumors at or beyond the second order biliary branches are classified as intrahepatic and make up the remaining 10%. Intrahepatic CCA (ICC) are predominantly adenocarcinoma with mucin production and a variable degree of differentiation [34].

**Figure 11:**
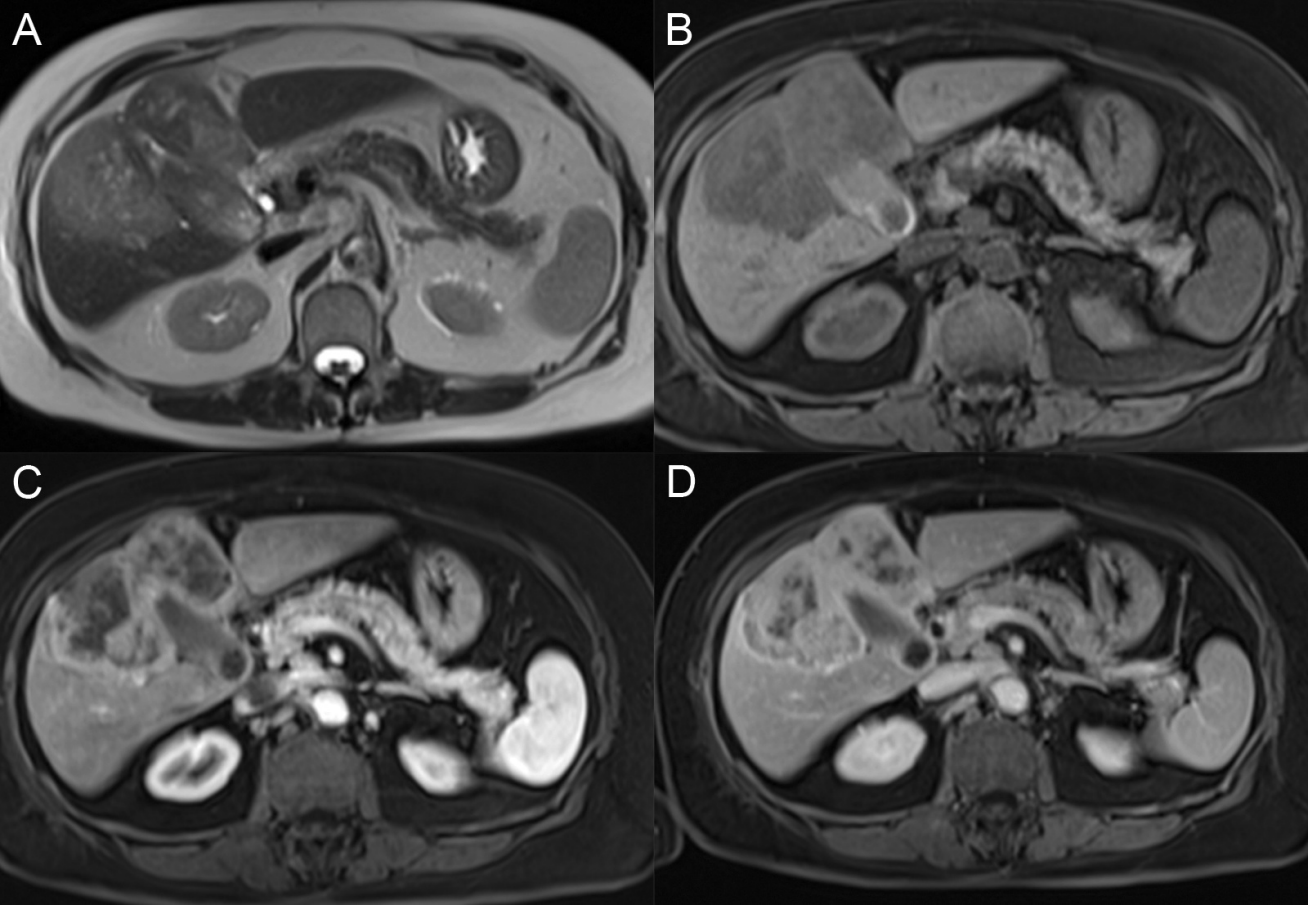
Intrahepatic cholangiocarcinoma A, B: The large mass in the right hepatic lobe has bright signal intensity on axial T2 and a dark signal on T1 fat saturation (FS) (B). C: The mass has a thick rind of peripheral avid enhancement on the axial T1 FS arterial phase. D: The mass enhancement is irregular and persistent on the axial T1 FS delayed phase.

**Figure 12:**
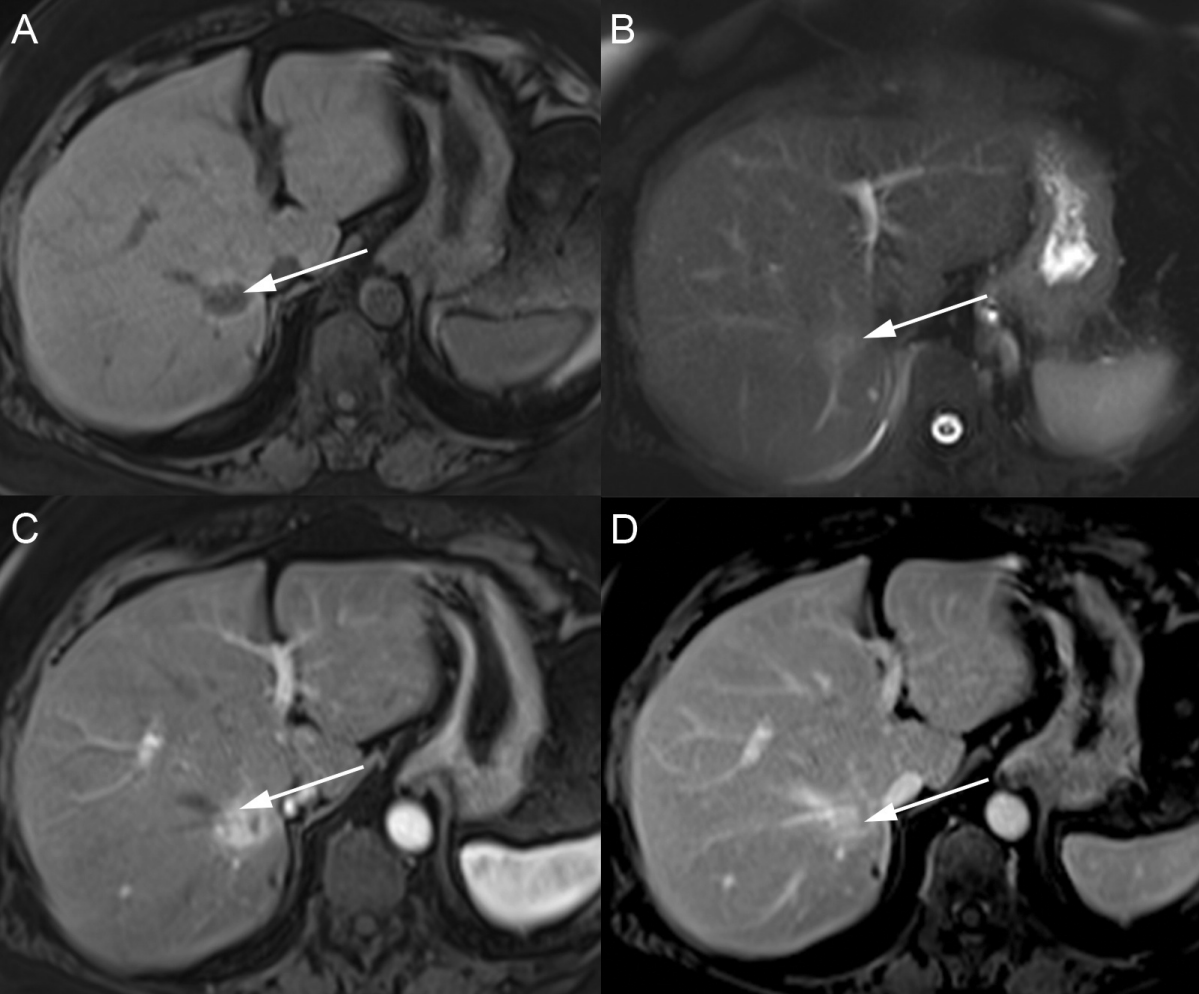
Intrahepatic cholangiocarcinoma A, B: The mass in the right hepatic lobe (arrows) has a dark signal intensity on axial T1 fat saturation (FS) (A) and a bright signal intensity on axial T2 FS (B). C: After contrast, the mass has avid enhancement on the axial T1 FS arterial phase. D: The mass enhancement persists on the axial T1 FS delayed phase (D).

Three growth patterns of ICC include mass-forming, periductal-infiltrating, and intraductal intrahepatic. Mass forming is the most common subtype and has a variable appearance at MRI scan depending on the degree of fibrosis and necrosis (Figures 11-12). Usually, the tumor is hypointense on T1W with variable peripheral T2W hyperintensity and central T2W hypointensity. Often, there are dilated biliary ducts peripheral to the lesion and overlying capsular retraction if the tumor is peripheral. Four enhancement patterns have been described. Most commonly, the mass has early peripheral arterial phase enhancement, which progresses centrally and washes out peripherally over time. This pattern should not be mischaracterized as a hemangioma because of the progressive nature. Other patterns include early peripheral enhancement with a nonenhancing central scar, a progressive complete enhancement, or complete arterial enhancement with washout [32]. Given the variable enhancement patterns, mass-forming ICC can mimic adenocarcinoma metastases and HCC, so tissue sampling is often needed to confirm the diagnosis.

Periductal-infiltrating and intraductal ICC growth patterns are associated with biliary tree findings rather than a discrete mass. The periductal-infiltrating ICC pattern results from tumoral growth along the bile ducts, resulting in diffuse periductal thickening and enhancement. Ducts may be dilated or narrowed, and this pattern is difficult to distinguish from benign strictures. Intraductal ICC has variable patterns of ductal dilation and stricture with or without papillary and/or polypoid intraluminal masses [32]. 

Combined Type HCC-CCA

**Figure 13:**
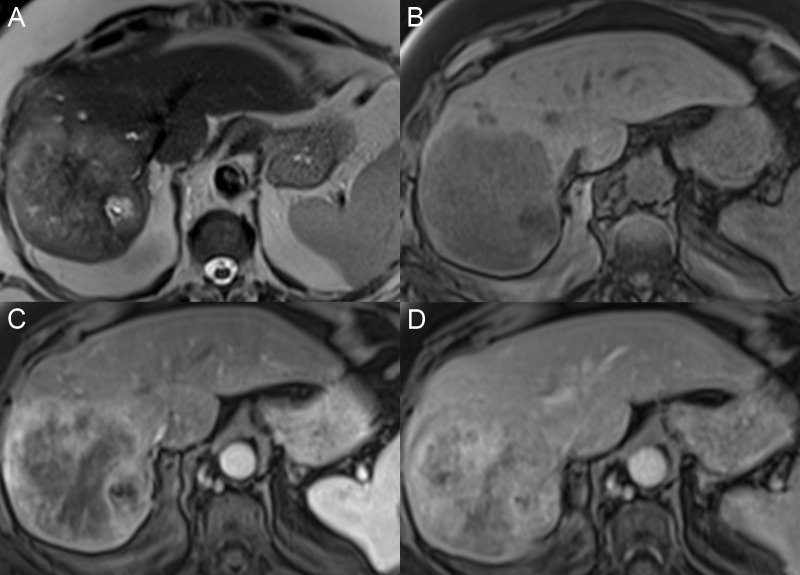
Combined hepatocellular and cholangiocarcinoma A: The large mass in the right hepatic lobe has a heterogneous bright signal on axial T2. B: The mass has a hypointense signal on axial T1 fat saturation (FS). C: After contrast, there is heterogeneous enhancement on the axial T1 FS arterial phase. D: The mass has progressive enhancement on axial T1 FS delayed images. Biopsy showed combined hepatocellular carcinoma and cholangiocarcinoma.

Combined type HCC-CCA is a rare primary liver malignancy that has the histological features of both HCC and ICC in patients both with and without a chronic liver disease. An initial description by Allen & Lisa (1949) reports three subtypes of tumors: 1) completely separate masses of HCC and ICC; 2) contiguous nodules of HCC and ICC that intermingle; and 3) a complete mixed mass of HCC and ICC. Given the mixed and variable distribution of the two neoplastic cell types, the imaging appearance can vary (Figure 13). Lesions may be peripheral with capsular retraction and are often T1W hypointense and T2W heterogeneous, usually without necrosis. The enhancement pattern can vary based on the dominant cell type, with peripheral arterial enhancement and variable delayed enhancement and washout [35]. A recent retrospective review of 32 patients with combined HCC-CCA post resection or transplantation found that those with > 50% HCC histology were more similar to HCC with arterial enhancement and washout, while those with > 50% ICC histology demonstrated delayed enhancement [36].

Rare Primary Hepatic Malignancies

Although liver malignancies arising from tissue other than hepatocytes and bile ducts are extremely rare, these tumors may have features that overlap with more common malignancies or have unique appearances that allow for precise diagnosis. Malignant vascular tumors include epithelioid hemangioendothelioma and hepatic angiosarcoma.

Epithelioid hemangioendothelioma

**Figure 14:**
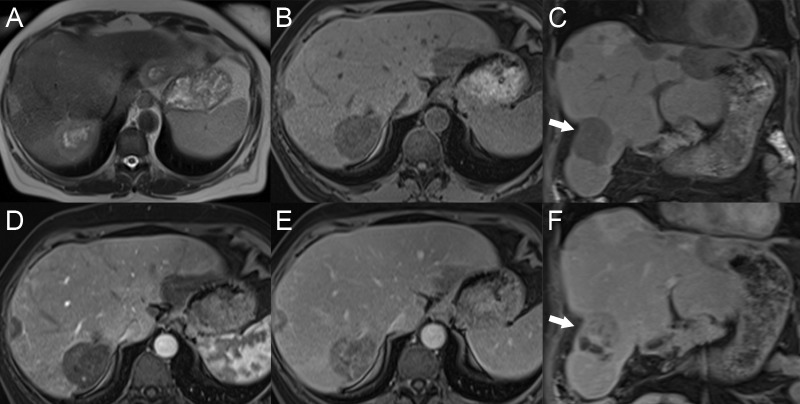
Epithelioid hemangioendothelioma A, B, C: Epithelioid hemangioendothelioma presents as multifocal masses with a bright signal on axial T2 (A) and dark on axial T1 fat saturation (FS) (B) and coronal T1 FS (C). D: After contrast, the lesions are hypoenhancing on the axial T1 FS arterial phase. E, F: The masses have gradual delayed enhancement on the axial (E) and coronal (F) T1 FS delayed phase. The segment 6 mass has overlying capsular retraction (arrows).

Epithelioid hemangioendothelioma is a low- to intermediate-grade malignant neoplasm that arises from the vascular endothelium with peripheral growth along the sinusoids and a variable dense central fibrosis. The tumor is composed of dendritic spindle cells and epithelioid round cells with an abundant mixoid matrix and fibrous stroma. Up to 30% may progress to some degree of sclerosis [37] (Figure 14). On MRI scan, the patient will often have multiple coalescent, peripheral liver nodules with capsular retraction. The tumor is T1W hypointense and has variable target-like T2W hyperintensity, with the brightest signal in the central portion and a T2W hypointense peripheral rim. After contrast, lesions have a thin rim of early enhancement with gradual, progressive enhancement to the center [38-40].

Primary hepatic angiosarcoma

**Figure 15:**
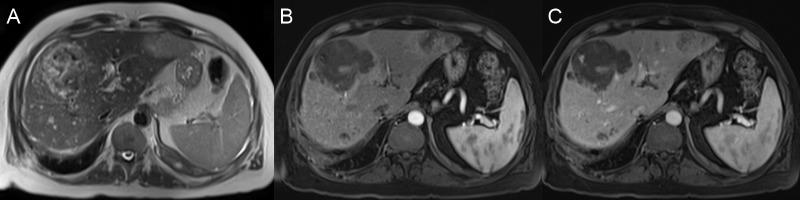
Hepatic angiosarcoma A: Large tumors in the right and left lobes have heterogeneous T2 signal with bright areas, but not as bright as hemangioma. B, C: After contrast, the masses have bizzare-appearing peripheral arterial enhancement on the axial T1 FS arterial phase (B) with gradual, heterogeneous enhancement on the axial T1 FS delayed phase (C).

Primary hepatic angiosarcoma is an aggressive vascular malignancy with very poor one-year survival. The tumor is composed of poorly organized vessels and cavities lined by malignant endothelial cells. Solid nodular areas composed of spindle cells may also form [37]. While these tumors are classically associated with exposure to toxins such as thorium dioxide (thorotrast), vinyl chloride, and arsenic, a recent multicenter review by Pickhardt et al. found 42% associated with cirrhosis [41]. This review of 35 patients at four centers imaged with CT and/or MRI scan is the largest cohort in current literature. Imaging features include large, multifocal masses involving both lobes of the liver. The lesions most commonly had small, heterogeneous areas of late arterial phase enhancement that progressed with variable patterns over time (Figure 15).

The lesions tended to have large hypovascular spaces without enhancement over the typical post-contrast duration. While some lesions have an enhancement pattern of delayed peripheral to central enhancement similar to hemangioma, these lesions had much more bizarre and disorganized enhancement and exhibited rapid growth over serial studies. Other lesions had the reverse pattern, enhancing from the center to the periphery over time. Masses compressed or displaced but did not invade the hepatic vasculature. Metastatic lesions were found in 45.7%, most commonly to the spleen, followed by the peritoneum, lungs, and pericardium.

Primary hepatic lymphoma

**Figure 16:**
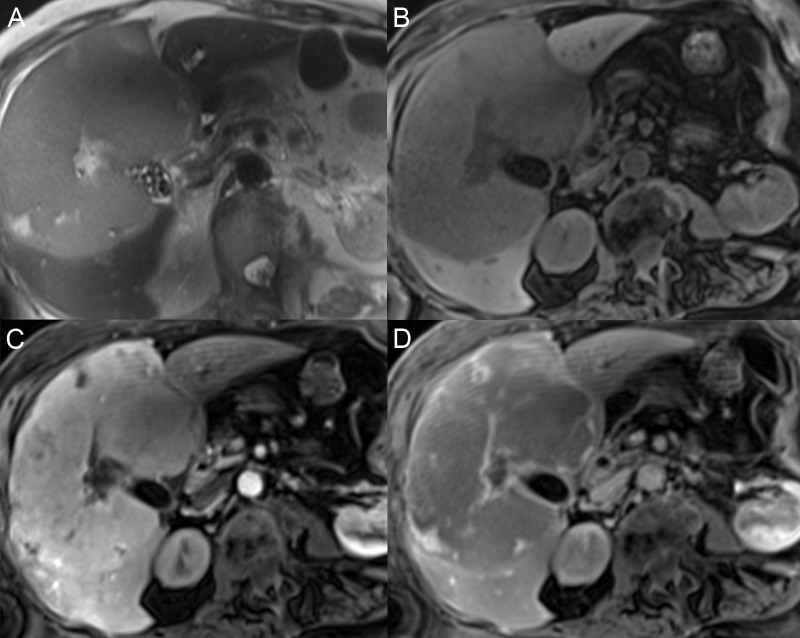
Solitary hepatic lymphoma A: The large mass filling the central liver has a mildly hyperintense signal on axial T2. B: The mass has a hypointense signal on axial T1 fat saturation (FS). C, D: After contrast, the mass has avid early enhancement on the axial T1 FS arterial phase (C) with washout on the axial T1 FS delayed phase (D). Biopsy confirmed the diagnosis of lymphoma.

**Figure 17:**
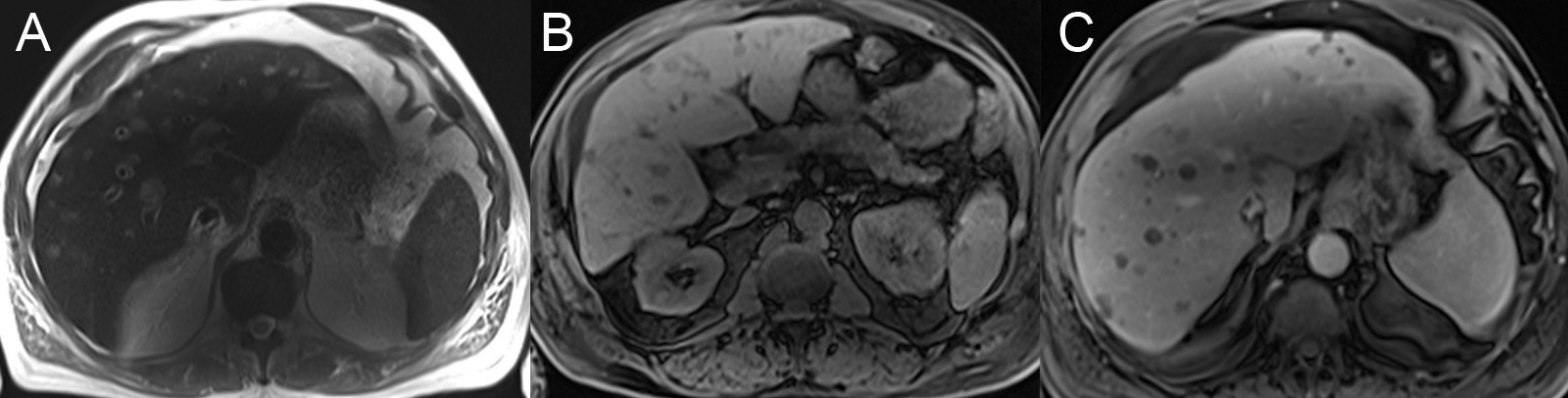
Multifocal hepatic lymphoma A: Multiple lesions throughout the liver with a hyperintense signal on axial T2. B: The masses have a hypointense signal on axial T1 fat saturation. C: After contrast, the masses have mild central targetoid enhancement on the axial T1 FS venous phase. Biopsy showed a B cell lymphoma.

The primary hepatic lymphoma accounts for less than 1% of all cases of lymphoma and occurs when lymphoma is confined to the liver, without disease in the lymph nodes, spleen, bone marrow, or other lymphomatous structures [42-43]. The majority of patients present with multiple lesions, though up to one third may have a solitary lesion. The MRI appearance of these lesions is nonspecific, as lesions have a T1 hypointense and T2 mildly hyperintense signal and enhance less than the adjacent hepatic parenchyma (Figure 16-17) [42,44]. More rarely, a diffuse, infiltrative pattern has also been described, which may present as an enlarged liver with indistinct lesions [42,45]. Another uncommon form of intravascular lymphoma grows along small vessels with resultant embolization of the hepatic arterial and portal venous branches with infiltrative tumor cells. Patient symptoms are related to primary liver involvement, including hepatomegaly, right upper quadrant pain, and abnormally elevated liver function tests.

## Conclusions

By understanding the MRI features of primary hepatic malignancies, these lesions can be appropriately included in the differential diagnosis of lesions on abdominal imaging studies. Recognizing a background chronic liver disease helps narrow the differential toward HCC and CCA. With good imaging techniques, biopsies can be avoided in patients whose lesions meet the imaging definition for HCC. An MRI scan can help determine which patients may be candidates for liver transplantation. More rare malignancies may not have definitive imaging features but should be considered in the correct setting, and awareness of these tumors allows for a more complete differential diagnosis.
